# Allopregnanolone: The missing link to explain the effects of stress on tic exacerbation?

**DOI:** 10.1111/jne.13022

**Published:** 2021-08-22

**Authors:** Marco Bortolato, Barbara J. Coffey, Vilma Gabbay, Simona Scheggi

**Affiliations:** ^1^ Department of Pharmacology and Toxicology College of Pharmacy University of Utah Salt Lake City UT USA; ^2^ Research Consortium on NeuroEndocrine Causes of Tics (ReConNECT); ^3^ Department of Psychiatry and Behavioral Science Miller School of Medicine University of Miami Miami FL USA; ^4^ Department of Psychiatry and Behavioral Sciences Albert Einstein College of Medicine Bronx NY USA; ^5^ Department of Molecular and Developmental Medicine School of Medicine University of Siena Siena Italy

**Keywords:** allopregnanolone, animal models, tics, Tourette’s disorder

## Abstract

The neurosteroid allopregnanolone (3α‐hydroxy‐5α‐pregnan‐20‐one; AP) elicits pleiotropic effects in the central nervous system, ranging from neuroprotective and anti‐inflammatory functions to the regulation of mood and emotional responses. Several lines of research show that the brain rapidly produces AP in response to acute stress to reduce the allostatic load and enhance coping. These effects not only are likely mediated by GABA_A_ receptor activation but also result from the contributions of other mechanisms, such as the stimulation of membrane progesterone receptors. In keeping with this evidence, AP has been shown to exert rapid, potent antidepressant properties and has been recently approved for the therapy of moderate‐to‐severe postpartum depression. In addition to depression, emerging evidence points to the potential of AP as a therapy for other neuropsychiatric disorders, including anxiety, seizures, post‐traumatic stress disorder and cognitive problems. Although this evidence has spurred interest in further therapeutic applications of AP, some investigations suggest that this neurosteroid may also be associated with adverse events in specific disorders. For example, our group has recently documented that AP increases tic‐like manifestations in several animal models of tic disorders; furthermore, our results indicate that inhibiting AP synthesis and signalling reduces the exacerbation of tic severity associated with acute stress. Although the specific mechanisms of these effects remain partially elusive, our findings point to the possibility that the GABAergic activation by AP may also lead to disinhibitory effects, which could interfere with the ability of patients to suppress their tics. Future studies will be necessary to verify whether these mechanisms may apply to other externalising manifestations, such as impulse‐control problems and manic symptoms.

## 1 INTRODUCTION

**FIGURE 1:**
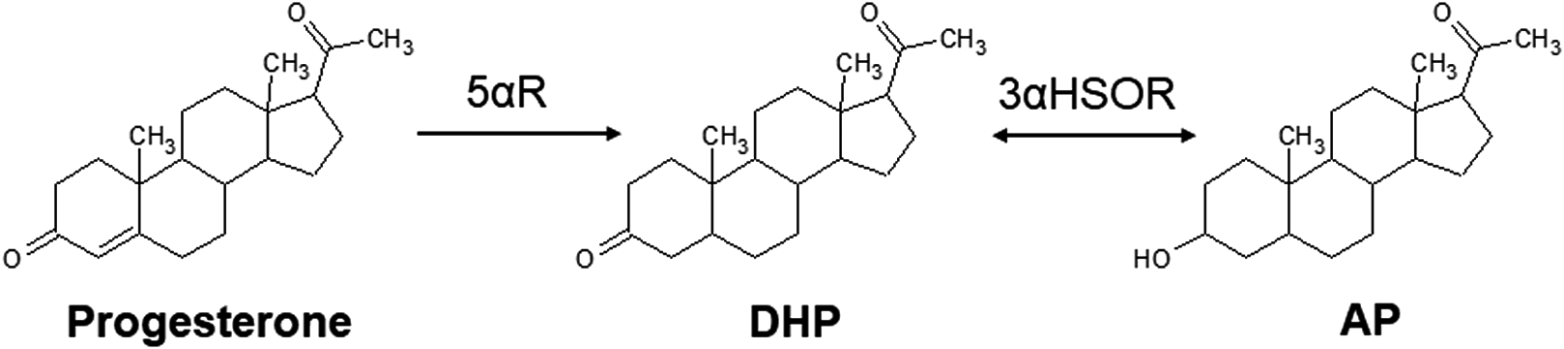
Allopregnanolone (AP) synthesis pathway. 5αR, 5α‐reductase; DHP, dihydroprogesterone; 3αHSOR, 3α‐hydroxysteroid oxidoreductase

The neurosteroid allopregnanolone (3α‐hydroxy‐5α‐pregnan‐20‐one; AP) is the product of a two‐step biosynthetic process from progesterone: the first step, catalysed by the enzyme 5α‐reductase (5αR), is the irreversible conversion of progesterone into 5α‐dihydro‐progesterone (DHP); the second step, mediated by 3α‐hydroxysteroid oxidoreductase (3α‐HSOR), is the reduction of DHP into AP^1^ (Figure 1).

This metabolic pathway is expressed in several brain areas implicated in emotional regulation, including the cortex and limbic regions, underscoring the role of this neurosteroid in affective modulation. In addition to this mechanism, the same two enzymes catalyse the synthesis of other neurosteroids, such as tetrahydrodeoxycorticosterone (3α,21‐dihydroxy‐5α‐pregnan‐20‐one; THDOC) and 3α‐androstanediol (5α‐androstane‐3α,17β‐diol).^2^

The best‐characterised mechanism of action of AP (as well as THDOC and 3α‐androstanediol) is the activation of GABA_A_ receptor,^3^ a chloride ion channel consisting of five subunits (out of 19 different subtypes: six α, three β, three γ and three ρ subunits, and one each of ϵ, δ, θ and π subunits). AP binds to two highly conserved sites within GABA_A_ receptors, localised within the transmembrane domains of α and β subunits, in a distinct position from the benzodiazepine site.^4^, ^5^ The strength and duration of the action of AP is also influenced by the subunit composition of GABA_A_ receptors. For example, AP enhances either the tonic or phasic inhibition mediated by these receptors, depending on the presence of δ or γ2 subunits, respectively.^6^, ^7^, ^8^, ^9^, ^10^ In addition to GABA_A_ receptor subunit composition, the effects of AP vary depending on its concentrations. In the nanomolar range, AP acts as a positive allosteric modulator by prolonging spontaneous chloride currents.^11^, ^12^ However, at concentrations higher than 10 μm (such as those that occur in the brain at the end of the pregnancy),^13^ AP acts as a GABA_A_ receptor agonist, and its effect is sufficient to suppress excitatory neurotransmission.^14^

The mechanisms of action of AP are not limited to GABA_A_ receptors. For example, low concentrations of AP activate several membrane progesterone receptors (mPRs).^15^ These G protein‐coupled, cell‐surface receptors are expressed in several brain regions, such as the limbic system, striatum, substantia nigra and cerebellum.^16^ The functional roles of mPRs are still poorly understood, although some of these receptors have been shown to influence GABA_A_ receptor signalling, by affecting its trafficking^17^ or facilitating the phosphorylation of β3 subunits.^18^, ^19^ Other mechanisms of action of AP include:

- the activation of pregnane‐X‐receptor,^20^ a nuclear receptor that controls the metabolism of xenobiotics.^21^ The interaction of AP with this receptor has been shown to mediate some of its neuroprotective and behavioural effects^20^, ^22^, ^23^;
- the positive modulation of P2X4 purinergic receptors^24^;
- the inhibition of nicotinic receptors^25^;
- the inhibition of toll‐like receptors 2, 4 and 7.^26^, ^27^

However, the specific contributions of each of these receptors to the behavioural effects of AP remain poorly understood.

Similar to the other GABA_A_ receptor activators, AP elicits potent sedative and anticonvulsant effects.^28^, ^29^, ^30^, ^31^, ^32^ Recent clinical data show that AP elicits potent antidepressant, and anxiolytic effects. Indeed, brexanolone (an exogenous analogue of AP) was recently approved by the US Food and Drug Administration for the treatment of postpartum depression,^33^ a condition associated with a physiological decline in progesterone and its metabolites,^34^, ^35^, ^36^ following the successful results of two multicentre, double‐blind, placebo‐controlled trials.^37^ Notably, several studies have documented a reduction in plasma and cerebrospinal AP levels of individuals affected by major depression.^38^, ^39^ Similar declines have been documented in anxiety and post‐traumatic stress disorder,^40^, ^41^, ^42^ potentially opening up the development of AP‐based treatments for these conditions. The beneficial effects of AP are not only limited to epilepsy and affective disorders, but also may extend to neurodegenerative disorders, likely given the well‐documented neurogenetic^43^ and neuroprotective properties of this neurosteroid.^44^, ^45^, ^46^, ^47^ In particular, several lines of research point to the therapeutic potential of AP for Alzheimer's disease.^48^, ^49^ Indeed, AP administration once a week for 6 months was found to promote neurogenesis, reduce β‐amyloid accumulation and improve memory and learning in one of the best‐validated animal models of Alzheimer's disease, the triple transgenic mouse.^50^

A detailed presentation of the therapeutic potential and applications of AP is beyond the scope of this article, although several excellent reviews are available.^32^, ^49^, ^51^, ^52^

Given these highly promising horizons, it may be tempting to regard AP as a panacea for a broad array of neuropsychiatric problems. Nevertheless, just as in the case of other endogenous compounds with therapeutic potential, caution should be advocated about overgeneralising the beneficial effects of AP. Although most research attention has been devoted to the therapeutic potential of AP and other neurosteroids, some emerging evidence, particularly in animal models, suggests that there may be another side of the coin. A poignant example of this concept is offered by the potential role of AP as a causal factor for dysphoria and negative mood in women with premenstrual dysphoric disorder (PMDD).^53^ This condition is characterised by a cluster of irritability, aggression, and emotional lability during the luteal phase of the menstrual cycle (when progesterone levels are exceptionally high).^54^ Although no consistent difference in AP levels has been shown between PMDD‐affected women and healthy controls,^55^, ^56^, ^57^ Timby et al^58^ reported that this condition is associated with alterations of AP sensitivity over the menstrual cycle. Indeed, pharmacological inhibition of 5αR by finasteride has been proposed as a potential remedy to mitigate symptoms in women with PMDD.^59^

Another critical question awaiting experimental verification concerns the applicability of AP to conditions that lie on the opposite side of depression along the affective spectrum, such as hyperthymia, hypomania and mania. Several studies have documented that treatment with canonical antidepressants, even in individuals with unipolar depression, significantly increases the risk of mania.^60^ To the best of our knowledge, no evidence is currently available on the potential liability of AP for these conditions; nevertheless, the possibility that AP may also increase the risk for this type of switch should not be regarded as beyond the realms of possibility. Indeed, ketamine, comprising another rapid, potent antidepressant treatment (albeit based on a completely different mechanism of action than AP), has been recently reported to cause affective switch to manic symptoms in bipolar patients,^61^, ^62^, ^63^ even though this untoward effect does not appear to apply to major depression.^64^

Against this background, work performed by our group has pointed to the possible implication of AP and other neurosteroids in the pathophysiology of tic disorders, a category of neurodevelopmental conditions characterised by rapid, non‐rhythmic movements or utterances, typically executed in a recurrent, patterned fashion.^65^ Below, we briefly summarise the clinical course and neurobiology of these disorders, as well as the body of evidence that supports a potential modulatory role of AP for tic severity. Finally, we discuss what putative mechanisms may underlie AP's implication in tic disorders and review how these processes may inform the development of new therapies for these and other related neuropsychiatric problems.

## 2 TIC DISORDERS

### 2.1 Clinical course and phenomenology of tics

Although approximately 20% of children exhibit isolated tics,^66^, ^67^ these manifestations are not pathological in the majority of cases. However, when executed in a chronic, pervasive fashion, tics limit functioning and can lead to significant disability, negatively impacting socioemotional adjustment, educational attainment and quality of life.^68^, ^69^ The *Diagnostic and Statistical Manual of Mental Disorders*, 5th edition, lists three tic disorders among the neurodevelopmental disorders, with onset before age 18 years, which are differentiated based on tic characteristics and duration criteria^65^:

- Tourette's disorder (TD), characterised by multiple motor tics and at least one vocal tic which have been present for more than 1 year;
- Persistent (chronic) motor or vocal tic disorder, characterised by either motor or vocal tics for more than 1 year;
- Provisional tic disorder, described by single or multiple tics for less than 1 year.

These diagnostic distinctions, however, do not likely reflect neurobiological differences. Indeed, it has been argued that tic disorders should be regarded as a pathological spectrum. In support of this idea, most cases of provisional tic disorder evolve into chronic tic disorders because they do not remit within 1 year.^70^ The most disabling tic disorder, TD, has a prevalence of 0.5%‐1% in the paediatric population^71^, ^72^, ^73^ with a marked male preponderance (male:female = 3‐4:1).^74^, ^75^ The personal burden of TD is complicated by the very high prevalence of comorbid psychiatric disorders, including attention‐deficit hyperactivity disorder (ADHD), obsessive‐compulsive disorder (OCD), anxiety and depression.^76^, ^77^, ^78^, ^79^, ^80^ Given this background, the current pharmacotherapies for TD remain highly unsatisfactory. The main pharmacological strategies for TD are dopaminergic antagonists/partial agonists and alpha 2 agonists,^81^ which are associated with inconsistent efficacy and multiple significant adverse effects, including dyskinesias, cognitive dulling and metabolic problems.^82^, ^83^ More recent clinical trials targeting the dopaminergic system, including dopamine agonists (pramipexole) and vesicular monoamine transporters (valbenazine and deutetrabenazine), have been disappointing.^84^, ^85^

The clinical course of TD follows a typical developmental trajectory, with onset of tics around 6 years of age, a gradual progression reaching lifetime peak tic severity around 10‐12 years^86^ and subsequent attenuation or remission^87^, ^88^; however, it is estimated that about 24% of TD patients continue to experience moderate to severe tics throughout adulthood.^89^ Aside from these diachronic changes in severity, tics wax and wane over the course of days and months. These fluctuations impact every phenomenological aspect of tics, namely number, frequency, intensity, complexity and interference in daily life.^90^ Although the biological causes of these fluctuations remain elusive, several lines point to environmental stress as a crucial influence for tic severity. For example, ample evidence has documented that tic severity is associated with the intensity of stressful life events.^91^, ^92^ This relationship has been confirmed by longitudinal analyses, which have documented that cumulative psychosocial stress predicts future tic severity.^93^ Furthermore, other studies have shown that tic severity is correlated with self‐report ratings of daily stress^94^ and recent negative events.^95^ Although these studies support the conventional framework that acute or short‐term stress has a detrimental impact on tic severity, more detailed analyses of this relationship have recently outlined a more complex picture. For example, tics may be particularly sensitive to specific types of stressors, such as overstimulation, intense emotional tension, frustration, fatigue and sleep loss.^96^, ^97^ Conversely, the Trier social stress test, which is an experimental task requiring participants to deliver a speech to an unsympathetic audience, was found to decrease, rather than increase, tic execution.^98^ These results indicate that the relationship between stress and tics is multifaceted and specific to individual environmental challenges.

A helpful framework to understand the source of complexity of the relationship between stress and tics requires discussion of premonitory urges, unpleasant sensations of tension and discomfort that precede tic execution and increase the drive to tic.^99^ The execution of tics relieves the negative feelings associated with premonitory urges. The behavioural model of tic maintenance^100^, ^101^ posits that tics are negatively reinforced insofar as they reduce the discomfort associated with premonitory urges. This perspective is supported by preliminary studies on the stress response in TD patients. In particular, several studies have documented that TD patients respond to acute stressors with a magnified activation of the hypothalamic‐pituitary‐adrenal axis.^102^, ^103^ However, evening cortisol levels were negatively correlated with tic severity,^103^ suggesting that tics may be executed as a possible form of maladaptive stress coping in TD patients. In line with this perspective, several patients describe their tics as automatic or even voluntary reactions to suppress the premonitory urge.^104^ Although both urges and tics can be temporarily suppressed, this volitional control is generally stressful and aggravates urges up to a point in which tics become insuppressible. From this perspective, recent studies have shown that stress does not intrinsically increase tics, but rather impairs the ability to suppress them^105^ and control premonitory urges. These studies suggest that the relationship between stress and tic severity is likely influenced by a complex functional balance between the severity of premonitory urges and the ability to suppress tics.

An additional, yet critical dimension in this imbalance is the contribution of impulsivity. Several studies have shown that TD is characterised by an impairment of inhibitory control of behaviour.^106^ Recent studies have shown that, in TD patients, tic severity was correlated with waiting motor impulsivity, as tested with the four‐choice serial reaction time task.^107^ However, it should be noted that TD patients do not show greater impulsivity across all cognitive tasks,^108^ suggesting that specific domains of motor impulsivity may drive tics.

### 2.2 Neurobiological mechanisms of tics

**FIGURE 2:**
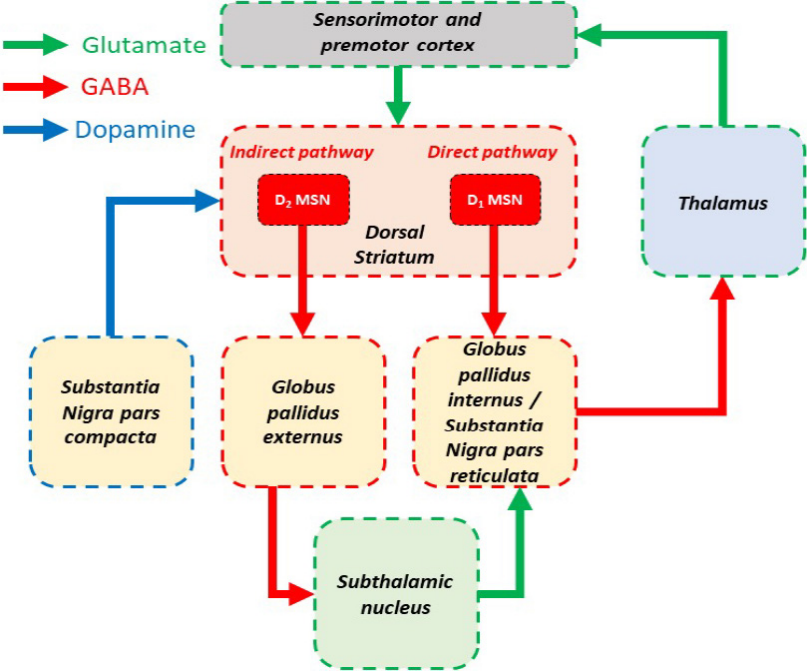
Schematic presentation of the cortico‐basal ganglia‐thalamo‐cortical (CBGTC) circuit. MSN, medium spiny neurone

Several lines of evidence indicate that tic disorders are underpinned by a broad set of anatomical and functional alterations within the cortico‐basal ganglia‐thalamo‐cortical circuitry (Figure 2).

In particular, structural imaging studies have documented that TD patients display a slight, yet significant, reduction of the volume of the dorsal striatum (caudate and putamen),^109^ as well as several compartments of the cortex.^110^, ^111^ Functional imaging studies have shown that tics are caused by a transient excess of activity of the connectivity between the cortex and the basal ganglia (and, in particular, the striatum).^112^ Tic execution is generally preceded by activation of the supplementary motor and anterior cingulate cortex, followed by stimulation of the putamen and the cerebellum.^113^, ^114^

The molecular and neurobiological causes of these alterations remain poorly understood, even though several studies have documented a selective loss in cholinergic and parvalbumin‐positive GABAergic interneurones in the dorsal striatum of individuals with severe TD.^115^, ^116^, ^117^ Building on this evidence, it is possible that a local reduction in striatal interneurones (likely a result of genetic and early‐life inflammatory factors) may lead to the formation of "focal disinhibition areas" in the dorsal striatum.^118^ In addition, several lines of research have shown a reduction in GABA content in the cortex of TD patients.^119^, ^120^ Another critical factor in tic ontogeny is the overactivation of dopaminergic neurotransmission in the nigrostriatal pathway,^121^, ^122^, ^123^, ^124^, ^125^ which may favour the emergence of off‐target movements by inhibiting the indirect pathway.^126^

The mechanisms of premonitory urges are less clear, although functional imaging studies suggest that these phenomena are driven by connectivity of the motor cortex, insula and supplementary motor area.^127^, ^128^, ^129^ Overall, these data highlight that premonitory urges and other sensory antecedents of tics are based on the activation of cortical regions involved in the modulation of sensory processing and motor output.

Of relevance to the present discussion, several studies have shown that tic suppression and cognitive control of motor behaviour are underpinned by the activation of the prefrontal cortex (PFC).^130^, ^131^, ^132^ Interestingly, the relationship between tic severity and waiting impulsivity is mediated by connectivity between the orbitofrontal cortex (a subregion of the PFC particularly susceptible to the adverse effects of stress) and the caudate nucleus.^107^

### 2.3 Animal models of tic disorders

One of the best research tools for examining the functional and molecular substrates of tics is provided by animal models.^128^ However, a critical conceptual hurdle in modelling TD is that very few animals display spontaneous tic‐like behaviours with a compelling construct and predictive validity.^133^

One of the few mouse models that exhibits these responses is afforded by D1CT‐7 mice, a transgenic line harbouring a cholera toxin subunit in neurones expressing D_1_ dopamine receptors.^134^, ^135^ A synopsis of the phenotypes of D1CT‐7 and their relevance to TD and comorbid entities is provided in Table 1. These animals display short (0.05‐0.1 s) clonic bursts, highly isomorphic with simple tics. Additionally, D1CT‐7 mice also display other phenotypes reminiscent of ADHD and OCD, including hyperlocomotion and perseverative responses. In addition to this face validity, D1CT‐7 mice also carry a high degree of predictive validity, underscored by their sensitivity to hallmark therapies for TD, such as antipsychotics and clonidine.^136^, ^137^ D1CT‐7 mice respond to acute environmental stressors with a marked exacerbation of tic‐like behaviours. Specifically, we found that spatial confinement in a cylinder within the home cage leads to a substantial increase in tic‐like behaviours and prepulse inhibition (PPI) deficits. Both of these behavioural abnormalities are countered by benchmark therapies for TD, such as haloperidol and clonidine.^136^ Although their construct validity as a TD model was initially questioned,^137^ recent discoveries on tic ontogeny have documented that the origin of tic‐like responses is based on the same type of sensorimotor cortical hyperactivation observed in TD (a detailed discussion of this issue is provided elsewhere 133).

**TABLE 1:** Comparison of phenotypes in D1CT‐7 mice and Tourette's disorder (TD) patients

	Phenotypes in DICT‐7 mice	Phenotypes in TD patients
Face validity	Sudden axial jerks	Tics
	PPI deficits	PPI deficits
	Hyperlocomotion	Hyperactivity in ADHD (?)
	Increased perseverative behaviours (digging, rearing, grooming)	Complusions in OCD
	Stress‐induced exacerbation of jerks and repetitive behaviour	Stress‐induced exacerbation of tics
Construct validity	Neuropotentiation of somatosensory cortex	Hyperactivity of somatosensory cortex during urges
Predictive validity	Response to D_2_ receptor antagonists	Response to haloperidol and pimozide
	Response to D_2_ receptor antagonists	Response to ecopipam
	Response to clonidine	Response to clonidine

Abbreviations

ADHD, attention‐deficit hyperactivity disorder; OCD, obsessive‐compulsive disorder; PPI, prepulse inhibition.

Aside from the case of D1CT‐7 mice, several questions remain open on the heuristic criteria to define which behavioural abnormalities in rodents can be used to model tics.^138^ Models of focal disinhibition, generated by microinjections of GABA_A_ receptor antagonists (bicuculline and picrotoxin) in the dorsal striatum,^139^, ^140^ are critical for validating the causal implication of the proximal ontogenic mechanisms of tics because they also engage in rapid, tic‐like bursts of activation of isolated muscle groups. However, these models are not well suited for studying tic phenomenology or validate novel therapies because they are based only on striatal mechanisms rather than on the modulatory processes from other brain regions, such as the cortex, midbrain and cerebellum. A viable alternative is provided by spontaneous or pharmacologically induced stereotypies (and particularly grooming, digging, and rearing sequences). Just like tics, these responses are perseverative, purposeless, can be increased by dopaminergic agonists and environmental stress, and respond to most benchmark pharmacological therapies for TD. Another behavioural paradigm used to probe the biological foundation of tic disorders is offered by PPI, defined as the attenuation of the startle response that occurs when the eliciting stimulus is preceded by a weaker signal.^141^ PPI is generally used as an operational index of sensorimotor gating, namely the perceptual domain that enables the exclusion of irrelevant information.^142^ Several premises underscore the translational relevance of PPI with respect to TD: first, PPI deficits have been documented in TD patients^143^, ^144^, ^145^; second, the biological substrates of PPI overlap with the CBTCG circuitry^146^, ^147^; third, this index is impaired by dopaminergic agonists^148^, ^149^, ^150^ and reduced by antipsychotic medications^149^; and, fourth, PPI is impaired by environmental stressors in rodents and humans.^151^, ^152^

## 3 THE ROLE OF AP IN TIC DISORDERS

### 3.1 Clinical findings

Our first exploration of the potential involvement of neurosteroids in the ontogeny of tic disorders came from a single‐case observation in a 34‐year‐old man affected by severe, treatment‐refractory TD, characterised by explosive phonic tics, stereotyped coprolalia and self‐injuring motor tics, as well as cleaning and checking compulsions and contamination‐theme obsessions.^153^ Initially inspired by previous findings on the therapeutic effects of the antiandrogen flutamide in TD,^154^ we used the 5αR inhibitor finasteride (5 mg day^‐1^), which also exerts well‐recognised antiandrogenic properties by inhibiting the conversion of testosterone into the potent androgen dihydrotestosterone (DHT). Indeed, finasteride is currently approved for the therapy of conditions associated with high DHT levels, namely benign prostatic hyperplasia and male‐pattern baldness.^155^ We found that finasteride led to a gradual yet marked improvement of vocal and, to a lesser extent, motor tics, with no apparent adverse event other than a modest decline in libido. Over the course of 18 weeks, finasteride reduced total tic severity scores by approximately 38%. However, upon treatment discontinuation, the symptoms resumed abruptly, requiring rapid reinstatement of the therapy.^153^ This encouraging result led us to conduct a proof‐of‐concept open‐label study with 16 patients,^156^, ^157^ which confirmed our initial results and showed that the ameliorative effects of finasteride reached significance by week 6 of therapy, with a plateau by the week 12 week of finasteride administration. Importantly, our results documented that 81.2% of these patients reported that their tic reduction reflected their improved ability to suppress tics in stressful contexts. Similar results were reported in an independent pilot study conducted in Taiwan.^158^ Despite these encouraging results, our plans to conduct a double‐blind, placebo‐controlled trial were scuttled following emerging evidence on the increased risk of depression in a subset of patients treated with finasteride.^159^, ^160^

### 3.2 Preclinical findings

Our next step was to investigate the neuroanatomic and molecular substrates supporting the effect of finasteride in models of TD. To this end, we began testing the behavioural impact of this drug on the stereotypies and PPI deficits caused by non‐selective dopaminergic agonists in rats.^161^ Our findings showed that both finasteride and other 5αR inhibitors, such as dutasteride and SKF 105111, elicited potent antidopaminergic effects and reversed both stereotypies and PPI deficits induced by the dopaminergic agonists apomorphine and d‐amphetamine.^161^ These studies were followed by several experiments aimed at locating the neuroanatomical and molecular foundations of these effects. These follow‐up studies documented that the antidopaminergic effects of finasteride were supported by the PFC and the nucleus accumbens shell.^162^ Furthermore, we showed that finasteride specifically countered the effects of D_1_ (and possibly D_3_), rather than D_2_ dopamine receptors, both in rats and mice.^163^, ^164^ Interestingly, the findings of antidopaminergic properties of finasteride also led to the discovery of other potential therapeutic application of 5αR inhibitors in animal models of other motor disturbances, such as levodopa‐induced dyskinesias,^165^, ^166^ as well as in opioid use disorder.^167^ It is worth noting that the antidopaminergic effects of finasteride are not associated with extrapyramidal side effects, such as catalepsy,^161^ likely a result of the interference with D_1_, rather than D_2_ dopamine receptor signalling.

Recognising that the effects of finasteride in patients appeared to centre around their increased ability to suppress and camouflage tics in the presence of stress, we hypothesised that the mechanisms for finasteride might reflect the inhibition of the synthesis of AP and other neurosteroids implicated in the regulation of stress response, rather than DHT. This idea was also supported by the findings that the antipsychotic‐like effects of finasteride were present in both castrated male^162^ and female rats.

The most convincing demonstration of a primary role of AP in the regulation of PPI came from our analyses on the sensitivity of 5αR1 knockout mice to the PPI‐disrupting effects of D1 receptor agonists.^168^ Similar to finasteride‐treated animals, these mice exhibited no PPI deficits in response to the potent D1 receptor agonist SKF 82958^168^; however, these effects were fully restored following treatment with AP, but not other products of 5αR metabolism, indicating that this neurosteroid is necessary for the TD‐related effects of D1 receptor stimulation.^168^ To address whether a tic‐exacerbating stressor may also lead to TD‐related deficits through the up‐regulation of AP levels, we investigated the effects of sleep deprivation in PPI. Building on our discovery that sleep deprivation impairs sensorimotor gating,^169^ we documented that this manipulation increases 5αR expression in the PFC. Notably, we reported that sleep deprivation reduced PPI by increasing the concentration of AP in the PFC. Indeed, finasteride reversed these effects, whereas exogenous administration of AP exacerbated these deficits.^170^

Based on these findings, we investigated whether AP or other neurosteroids might be implicated in the ontogeny of tic‐like behaviours and gating deficits in D1CT‐7 mice. A synoptic view of the results of these experiments is reported in Table 2.

**TABLE 2:** Effects of neurosteroids and steroidogenesis inhibitors on tic‐like behaviours in D1CT‐7 mice

Neurosteroids/drugs	Effects on DICT‐7 mice
Progesterone	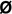
Dihydroprogesterone	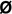
Allopregnanolone	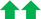
Tetrahydrodeoxycorticosterone	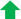
Isoallopregnanolone	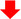
Testosterone	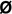
Dihydrotestosterone	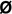
Finasteride (5αR inhibitor)	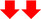
Dutasteride (5αR inhibitor)	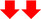
Indomethacin (3α‐HSOR inhibitor)	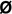

Our results showed that, in this model, acute stress led to a generalised enhancement of the levels of progesterone, DHP and AP in the PFC. We investigated the systemic effects of these steroids in TD but found that only AP elicited behavioural abnormalities akin to those observed following spatial confinement.^171^ Notably, D1CT‐7 mice were found to have higher baseline levels of AP in the PFC compared to their wild‐type controls; however, they did not show any significant change in the subunit expression of GABA_A_ receptors in this area. Furthermore, the 5αR inhibitor finasteride normalised behavioural alterations induced by stress in D1CT‐7 mice, without producing any such effects in wild‐type littermates.^171^ Notably, the same results were observed using the endogenous antagonist of AP, isoallopregnanolone (3β‐hydroxy‐5α‐pregnan‐20‐one, a natural 3β epimer of AP).^172^ Although isoallopregnanolone has an efficacy comparable to that of finasteride and does not elicit extrapyramidal symptoms, it does not produce the same profound depressogenic‐like effects observed after finasteride treatment.^173^ These data indicate that, unlike finasteride, isoallopregnanolone may be a viable therapy for reducing the adverse effects of acute stress on tic exacerbation.

### 3.3 Mechanisms of AP in tic exacerbation

At present, the downstream mechanisms by which AP exacerbates tic‐like behaviours and impairs PPI in rodent models of TD remain unclear. AP exerts a broad array of modulatory effects on dopaminergic transmission and signalling, which may help explain some of the effects observed in animal models of TD. For example, AP prevents the increase in extracellular dopamine concentrations induced by footshock stress^174^ but dose‐dependently increases dopamine release in the nucleus accumbens both in relation to baseline conditions and in response to morphine, a potent rewarding stimulus.^175^ This action is particularly notable because it may help explain previous data indicating that AP promotes motivated and reward‐directed responses^176^, ^177^ and reinstates ethanol‐seeking behaviour.^178^, ^179^ Given that the actions of AP on dopamine appear to be state‐dependent, it will be essential to verify whether these effects differ between animal models of TD and their controls, under normal conditions or in the presence of stress.

Our data also point directly to a selective effect of AP on the signalling of D1 receptors. Although ongoing studies are focusing on the molecular details of this interaction, it should be noted that, in line with our results, previous studies have also documented that AP modulates some behavioural effects of D1 receptor activation,^180^, ^181^ and both progesterone and AP affect the phosphorylation of DARPP‐32 (dopamine and cAMP‐regulated phosphoprotein of molecular weight 32 000), a critical neuronal phosphoprotein that integrates signalling information in dopaminoceptive neurones.^182^, ^183^

**FIGURE 3:**
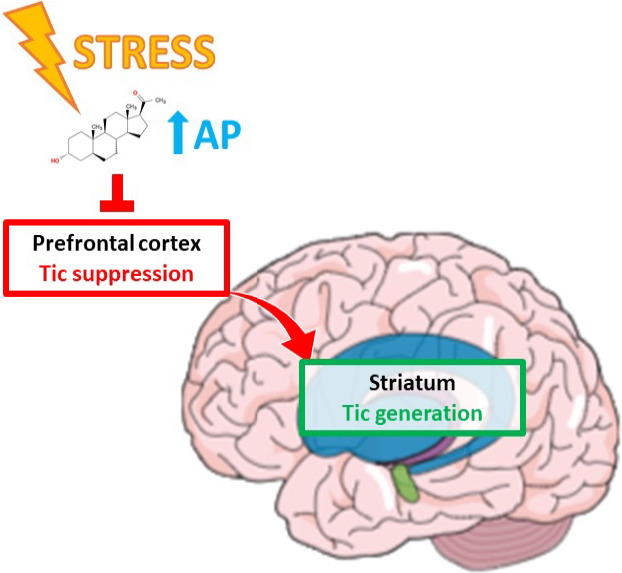
Schematisation of the role of allopregnanolone (AP) in the adverse effects of stress on tic suppression. Additional details are provided in the main text

Irrespective of the specific interaction with D1 receptors, the behavioural outcomes of AP are likely a result of the positive allosteric modulation of GABA_A_ receptors in the PFC. However, it should be noted that our experiments showed that, unlike the genetic inactivation of 5αR1, neither the GABA_A_ antagonist bicuculline nor the genetic knockout for GABA_A_ δ subunit affected the ability of D1 receptor agonist to impair PPI.^168^ These studies suggest that other receptors may be implicated in the effects of AP. Of note, neurosteroidogenic enzymes are co‐localised with GABA_A_ receptors in cortical pyramidal neurones; thus, high concentrations of AP in these cells may lead to aberrant inhibition of projection neurones in the PFC, resulting in greater stimulation of the striatum. Accordingly, stress has been shown to impair the function of the PFC.^184^ This framework would posit that AP may reduce the inhibitory connectivity of the PFC on the striatum, ultimately countering the mechanism of volitional tic suppression and facilitating tic execution (Figure 3). Alternatively, AP may be sulfonated into AP sulfate, which acts as a negative allosteric modulator of NMDA glutamate receptors.^185^

## 4 CONCLUSIONS AND FUTURE DIRECTIONS

The data summarised in this review show that converging lines of evidence support the implication of AP (and possibly other neurosteroids) in the pathophysiology of tic disorders. The most convincing data from our group suggest that AP may mediate the adverse effects of acute stress on tic severity and, possibly, contribute to the fluctuations in TD symptoms by modulating the ability of the PFC to inhibit the generation of tics in the dorsal striatum. Although this process provides a compelling explanation of the observed effects of finasteride in TD patients, future endocrinological and neuroimaging studies are warranted to verify how AP modifies tic suppression and its underlying neural patterns. From a therapeutic perspective, these ideas may lead to novel treatments aimed at stabilising AP levels in tic disorders. The demasculinising and depressogenic effects of finasteride raise significant concerns about its application as a therapy in children, particularly given consideration of the high comorbidity of depression and anxiety in TD patients; however, our recent data on isoallopregnanolone as a potential therapy with similar effectiveness as finasteride^172^ in mouse models of TD highlight that this endogenous AP antagonist (or other compounds with a similar mechanism of action) may be a promising therapeutic alternative for TD, given its optimal profile of clinical tolerability and safety.^186^

Another critical question that will need to be addressed by future investigations concerns the possibility that the processes by which AP can exacerbate tics may apply to other neuropsychiatric conditions, and in particular impulse‐control problems, given the notable neurobiological overlap between mechanisms of motor impulsivity and tic control.^107^ This possibility is indirectly supported by preliminary data indicating that finasteride reduces impulsivity^173^ and pathological gambling.^157^ In addition, we recently documented that finasteride also potently reduces opioid self‐administration,^167^ another behavioural response highly influenced by impulsivity. Assuming that AP can reduce the prefrontal control of striatal outputs, this mechanism may also be responsible for a disinhibitory effect, which may account for the exacerbation of impulsive behaviours in response to acute stress. From this perspective, it is worth noting that other GABA_A_ receptor activators, such as benzodiazepines, are occasionally associated with a significant increase of impulsive and externalising behaviour in vulnerable individuals, including children.^187^ These disinhibited reactions, such as hyperactivity, sexual disinhibition, hostility and rage, are also observed in response to other GABAergic sedatives, such as alcohol. These paradoxical reactions may reflect differences in GABA_A_ receptor sensitivity or other neurobiological differences in inhibitory control. From this perspective, it is interesting to note that the effects of AP on externalising behaviour may vary depending on the endogenous content of this neurosteroid. For example, in dominant male mice, low doses of AP increase aggression by approximately 50%^188^, ^189^; conversely, AP has anti‐aggressive effects in the mouse model of social isolation, which is accompanied by a dramatic decline of AP brain levels.^190^, ^191^

Building on these premises, we hypothesise that, in subjects with high baseline AP concentrations, this neurosteroid may promote externalising and impulsive reactions to acute stress, which may be particularly problematic in the presence of other predisposing factors. Conversely, in individuals with low endogenous levels of AP, its use may help reduce internalising responses to stress (such as depressive and anxious symptoms) by promoting euthymia and eudaimonia. This framework posits that AP levels in the brain may contribute to the dynamic of internalising and externalising styles of the stress response. If supported by experimental data, such a conceptualisation may point to a much more complex role of this neurosteroid in mood and personality regulation. More importantly, this direction may pave the way to a new generation of neurosteroid‐based therapies aimed at reattuning our corticolimbic responses to stress and reward.

## AUTHOR CONTRIBUTIONS

**Marco Bortolato:** Conceptualisation; Funding acquisition; Writing – review & editing. **Barbara J Coffey:** Writing – review & editing. **Vilma Gabbay:** Writing – review & editing. **Simona Scheggi:** Writing – review & editing.

### PEER REVIEW

The peer review history for this article is available at https://publons.com/publon/10.1111/jne.13022.

## Data Availability

Data sharing is not applicable to this article because no new data were created or analysed in this manuscript
